# DCE-MRI to distinguish all monoclonal plasma cell disease stages and correlation with diffusion-weighted MRI/PET-based biomarkers in a hybrid simultaneous whole body-2-[18F]FDG-PET/MRI imaging approach

**DOI:** 10.1186/s40644-024-00740-5

**Published:** 2024-07-11

**Authors:** Bastien Jamet, Hatem Necib, Thomas Carlier, Eric Frampas, Juliette Bazin, Paul-Henri Desfontis, Aurélien Monnet, Caroline Bodet-Milin, Philippe Moreau, Cyrille Touzeau, Francoise Kraeber-Bodere

**Affiliations:** 1 Nantes Université, Univ Angers, CHU Nantes, INSERM, CNRS, CRCI2NA, Médecine Nucléaire, F-44000 Nantes, France; 2 Nantes Université, Univ Angers, CHU Nantes, INSERM, CNRS, CRCI2NA, Radiologie, F-44000 Nantes, France; 3 Siemens, France; 4 Nantes Université, Univ Angers, CHU Nantes, INSERM, CNRS, CRCI2NA, Hématologie, F-44000 Nantes, France; 5 Nuclear Medicine Department, University Hospital, 1 Place Alexis Ricordeau, 44093 Nantes, France

**Keywords:** Multiple myeloma, Plasma cell dyscrasias, Multiparametric magnetic resonance imaging, Positron-emission tomography imaging

## Abstract

**Background:**

Dynamic contrast-enhanced-MRI (DCE-MRI) is able to study bone marrow angiogenesis in patients with multiple myeloma (MM) and asymptomatic precursor diseases but its role in the management of MM has not yet been established. The aims of this prospective study was to compare DCE-MRI-based parameters between all monoclonal plasma cell disease stages in order to find out discriminatory parameters and to seek correlations with other diffusion-weighted MRI and positron emission tomography (PET)-based biomarkers in a hybrid simultaneous whole-body-2-[18F]fluorodeoxyglucose (FDG)-PET/MRI (WB-2-[18F]FDG-PET/MRI) imaging approach.

**Methods:**

Patients with newly diagnosed Monoclonal gammopathy of undetermined significance (MGUS), smoldering multiple myeloma (SMM) or symptomatic MM according to international myeloma working group and underwent WB-2-[18F]FDG-PET/MRI imaging including bone marrow DCE sequences at the Nantes University Hospital were prospectively enrolled in this study before receiving treatment.

**Results:**

One hundred and sixty-seven patients (*N* = 167, mean age: 64 years ± 11 [Standard deviation], 66 males) were considered for the analysis. DCE-MRI-based Peak Enhancement Intensity (PEI), Time to PEI (TPEI) and their maximum intensity time ratio (MITR: PEI/TPEI) values were significantly different between the different monoclonal plasma cell disease stages, PEI values increasing and TPEI values decreasing progressively along the spectrum of plasma cell disorders, from MGUS stage to symptomatic multiple myeloma. PEI values were significantly higher in patients with diffuse bone marrow involvement (either in PET or in MRI images) than in those without diffuse bone marrow involvement, unlike TPEI values. PEI and TPEI values were not significantly different between patients with or without focal bone lesions.

**Conclusion:**

Different DCE-MRI-based parameters (PEI, TPEI, MITR) could significantly differentiate all monoclonal plasma cell disease stages and complemented conventional MRI and PET-based biomarkers.

## Introduction

Monoclonal gammopathy of undetermined significance (MGUS) occurs in 3.2% of people aged 50 years or older and in 5.3% of people aged 70 years or older [1]. MGUS consistently precedes smouldering multiple myeloma (SMM) and symptomatic multiple myeloma (MM) which are the different sequential stages of monoclonal plasma cell disease [2]. In the latest international myeloma working group (IMWG) imaging guidelines, both whole-body MRI (WB-MRI) and 2-[18F]fluorodeoxyglucose (FDG)-positron emission tomography (PET) with computed tomography (CT) imaging techniques can be performed at initial MGUS/SMM/MM workup [3, 4] in order to detect bone disease which is a criterion for starting therapy [5]. Thus, the combination of these two imaging modalities in a single simultaneous WB-2-[18F]FDG-PET/MRI scan seems attractive and preliminary data concerning diagnostic performance of this technique have been reported recently [6]. Dynamic contrast-enhanced-MRI (DCE-MRI) is able to study bone marrow microcirculation/angiogenesis in patients with MM and asymptomatic precursor disease [7, 8]. DCE-MRI has shown important prognostic value at baseline in SMM and symptomatic MM patients [9, 10] but its role in the management of MM patients has not yet been established. DCE-MRI could then be implemented into simultaneous WB-2-[18F]FDG-PET/MRI imaging in order to complement the different PET and MR-based diagnostic and prognostic data. Several attempts have been made to compare DCE-MRI-based parameters in different groups of MM and asymptomatic precursor disease but these have had limitations, either in omitting SMM patients [11] or in having a very small cohort [12]. In addition, the relationship between DCE-MRI-based parameters reflecting angiogenesis to other diffusion-weighted MRI and PET-based biomarkers such as apparent diffusion coefficient (ADC) reflecting bone marrow cellularity or standardized uptake value (SUV) reflecting clonal plasma cell morphology [13] has not been reported until now.

The aims of this prospective study was to compare DCE-MRI-based parameters between all stages of monoclonal plasma cell disease in order to identify discriminative parameters (primary objective) and to seek correlations with other diffusion-weighted MRI and PET-based biomarkers (secondary objective) in a hybrid simultaneous WB-2-[18F]FDG-PET/MRI imaging approach.

## Materials and methods

### Patients

**Fig. 1:**
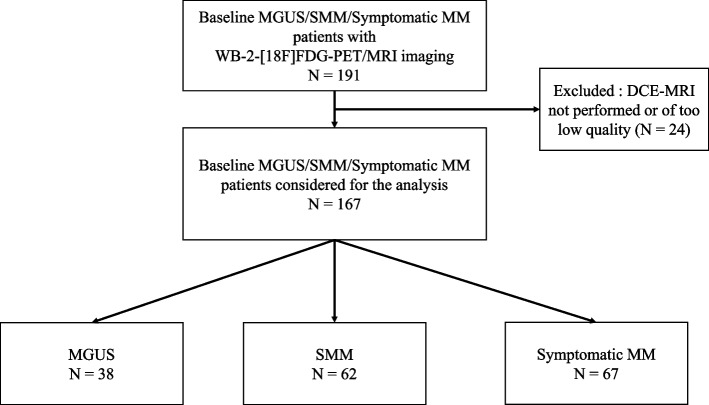
Flow Diagram. MGUS: monoclonal gammapathy of undetermined significance; SMM: smoldering multiple myeloma; DCE-MRI: dynamic contrast enhanced-MRI

This prospective study was approved by the local institutional review board committee. Informed consent was obtained from all individual participants included in the study. Patients with newly diagnosed MGUS, SMM or symptomatic MM according to IMWG criteria [5] and underwent WB-2-[18F]FDG-PET/MRI imaging including DCE sequences prior to treatment at the Nantes University Hospital were prospectively enrolled in this study (Flow Diagram, Fig. 1). Patients with MM were considered as symptomatic before the WB-2-[18F]FDG-PET/MRI imaging if any one or more myeloma defining event (MDE) were present: end organ damage attributed to the MM; hypercalcemia, renal insufficiency, anemia, osteolytic bone lesions and/or biomarkers of malignancy: clonal bone marrow plasma cell percentage ≥ 60%, involved/uninvolved serum free light chain ratio ≥ 100). If no MDE was present at baseline before the WB-2-[18F]FDG-PET/MRI imaging, patients were regarded as SMM. MGUS patients had serum monoclonal protein values < 30 g/L and clonal bone marrow plasma cells values < 10%. According to IMWG criteria [5], MGUS and SMM patients before the WB-2-[18F]FDG-PET/MRI imaging who displayed at least two unequivocal focal bone lesions (FLs) of size > 5 mm on MRI were reclassified as symptomatic MM.

### DCE-MRI protocol and other WB-2-[18F]FDG-PET/MRI-based parameters considered

DCE-MRI of the bone marrow was performed as previously proposed [11] by injecting a bolus of gadolinium contrast agent bolus of 0.2 ml/kg at an injection rate of 3–5 ml/s. This protocol consists of a 3D acquisition repeated every 3.1 s using a 3D Gradient Echo sequence (slice thickness 2 mm, FOV 300 mm, matrix 192, TR/TE: 2.32/0.72 ms, flip angle = 12◦, 40 repetitions) of the lumbar spine and the sacrum. Imaging was performed using a 3 T Biograph mMR (Siemens). The rest of the WB-2-[18F]FDG-PET/MRI imaging protocol has been described in detail elsewhere [6]. Briefly, diffuse bone marrow involvement (BMI) on PET images was defined as an uptake (homogeneous or heterogeneous) in the axial and appendicular skeleton higher than liver uptake. Maximal standardized uptake value (SUV_max_) of bone marrow (SUV_max_BM) was measured with regions of interest (ROIs) of similar size (4 cm^3^) placed within the vertebral body of L4 or L5 on a single slice (preferably sagittal), excluding FL if present at that level in order to consider only bone marrow. On MRI images, diffuse BMI was considered when there was a diffuse decreased signal on T1-in phase-DIXON weighted and diffuse increased signal throughout the marrow (relative to normal adjacent muscle) on high b-value images [14]. Mean values of apparent diffusion coefficient of the bone marrow (ADC_mean_ bone marrow) were registered as for PET imaging with ROIs placed within the vertebral body of L4 or L5, excluding FL if present at that level. True positivity of detected FL was confirmed with follow-up scans.

All PET and MRI images were interpreted and analyzed by nuclear medicine physicians and radiologists with special expertise in osteo-articular and especially MM imaging.

### DCE-MRI analysis

Image post-processing and DCE-MRI analyses were performed according to the semi-quantitative method previously described [11, 12]. An in-house 3DSlicer-plugin (Slicer.org, [15]) was used to extract the perfusion-based parameters measurement. Systematic ROIs represented by a sphere of 15 mm in diameter were manually drawn in order to study bone marrow within the vertebral body of L4 or L5 excluding FL or degenerative changes when present at this level.

**Fig. 2:**
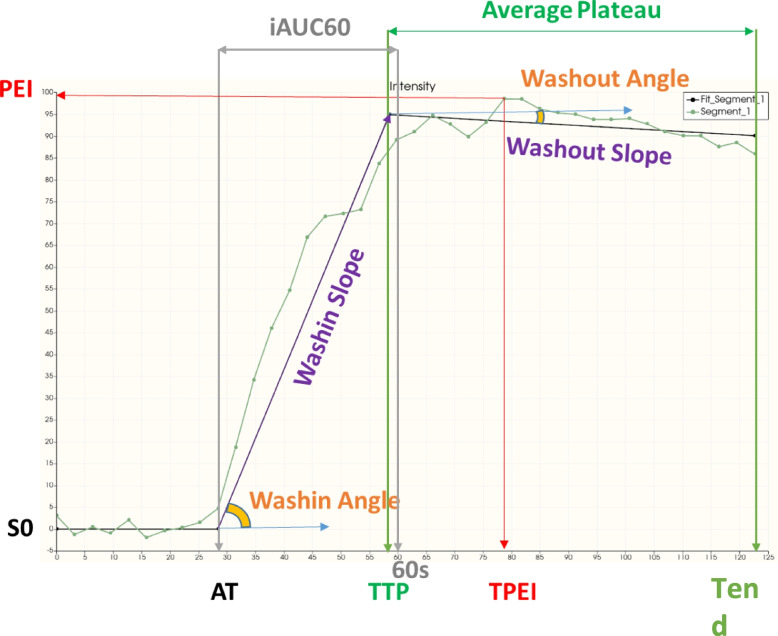
Time-signal intensity curve (TIC) obtained from DCE with all the measurement points studied giving different metrics that qualitatively characterize the DCE: S0 = First values signal (baseline), AT = Arrival Time, Smax = Maximum Signal, PEI = Peak Enhancement Intensity (Smax—S0), PER = Peak Enhancement Ratio (PEI/S0), TPEI = Time to PEI (Tmax -AT), TTP = Time To Peak (end of Wash-in—AT), T90 = Time before 90% of PEI, MITR = Maximum Intensity Time ratio (PEI/TPEI), nMITR = normalized MITR (100*PEI/(S0*TPEI)), WinSlope = up-slope (Wash-in), WinAngle = up-angle, WoutSlope = down-slope (Wash-out), WoutAngle = down-angle, SER = (Smax- S0)/(Send—S0), iAUC60 = AUC between AT and 60 s, AvgPlateau = AUC between TTP and Tend

The time-signal intensity curves (TICs) of DCE were then fitted and normalized to automatically derive semi-quantitative parameters. Figure 2 summarizes all the parameters obtained from the TIC. Note the difference between TPEI (Time to Peak enhancement intensity) and TTP (Time to Peak). TTP is the time from the arrival time to reach the highest value in the tissue during the Wash-in slope whereas TPEI is the time from arrival to reach the highest value in the TIC (not necessarily the same point).

### Statistical analysis

The distribution mean values of all parameters was compared between MGUS, SMM and symptomatic MM patients using Dunn’s test if the initial Kruskall-Wallis test was significant for multiple testing. *P*-value correction for multiple testing was performed using Holm–Bonferroni corrections. Correlation analyses between DCE-MRI-based and diffusion-weighted PET/MRI-derived biomarkers were performed using Wilcoxon rank-sum tests. Kendall's rank correlation coefficients with 95% confidence intervals (CIs) were calculated for correlation analyses between clinico-biological and TIC-derived parameters. A 5% significance level was used for all statistical tests. All statistical analyses were performed with R version 4.3.1 (http://www.R-project.org).

## Results

### Patients

One hundred and ninety-one patients underwent WB-2-[18F]FDG-PET/MRI imaging between May 2021 and May 2023. In twenty-four patients, DCE-MRI was not performed or was of too low quality (absence or very low aortic enhancement), leaving one hundred and sixty-seven patients (*N* = 167) for final analysis (Flow Diagram, Fig. 1). Among them, and considering WB-2-[18F]FDG-PET/MRI imaging results (especially the presence of at least two unequivocal FLs of size > 5 mm on MRI), thirty-eight (23%) had MGUS, 62 (37%) had SMM and 67 (40%) had symptomatic disease as defined above. Table 1 reports the patient’s clinical and biological characteristics. Among symptomatic MM patients, 15% had hypercalcemia, 20% renal insufficiency, 57% anemia. Six of the 67 patients had only one biomarker of malignancy (involved/uninvolved serum free light chain ratio ≥ 100) without end organ damage attributed to the MM.

**Table 1:** Patient’s clinical and biological characteristics

Variable	Symptomatic MM *n* = 67	SMM *n* = 62	MGUS *n* = 38
Mean age ± SD	64.2 ± (9.9)	66 ± (12.5)	61.8 ± (10.6)
Male	25/67 (37%)	24/62 (39%)	17/38 (45%)
R-ISS I	26/67 (39%)	N/A	N/A
R-ISS II	23/67 (34%)	N/A	N/A
R-ISS III	18/67 (27%)	N/A	N/A
Type of monoclonal Ig or free lights chains			
IgGκ	18/67 (27%)	23/62 (37%)	14/38 (37%)
IgGλ	7/67 (10%)	19/62 (31%)	9/38 (24%)
Κ	12/67 (18%)	4/62 (6%)	2/38 (5%)
IgAλ	9/67 (13%)	3/62 (5%)	5/38 (13%)
Λ	8/67 (12%)	3/62 (5%)	4/38 (10.5%)
IgAκ	13/67 (20%)	10/62 (16%)	4/38 (10.5%)
Mean serum monoclonal Ig (g/L) ± SD	28.6 ± (19.5)	23.2 ± (15)	11.5 ± (8.6)
Mean bone marrow plasma cells (%) ± SD	28 ± (25)	17 ± (15)	4 ± (2.85)

*MM* multiple myeloma, *R-ISS* revised international staging system, *N/A* not applicable, *SD* standard deviation

### DCE-MRI-based parameters in different monoclonal plasma cell disease stages

**Fig. 3:**
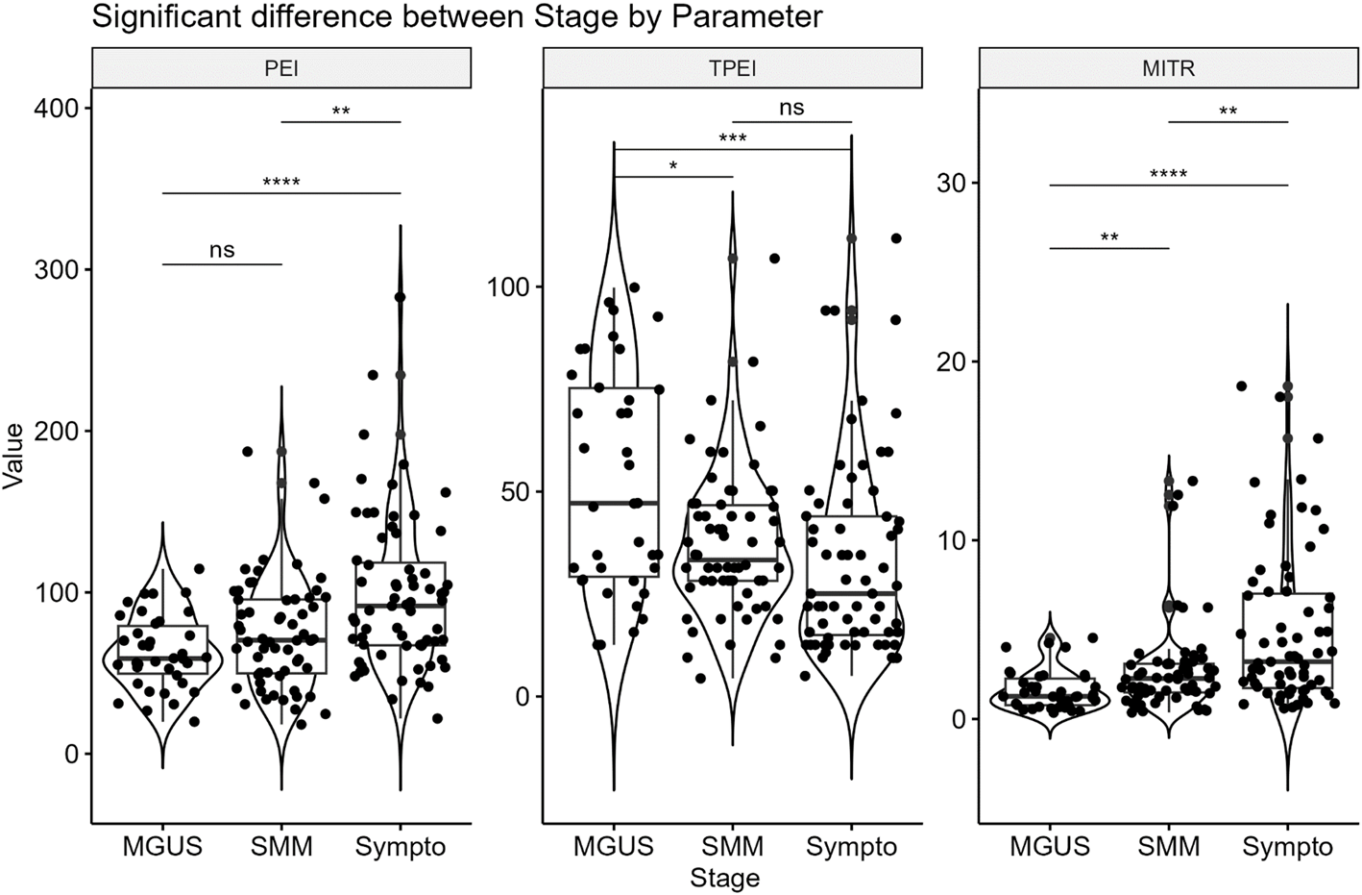
DCE-MRI-based PEI,TPEI,MITR parameters in different monoclonal plasma cell disease stages. NS: no significant; “*”: p < 0,05; “**”: p < *10*^*–2*^; “***”: p < *10*^*–3*^; “****”: p < *10*^*–4*^*)* PEI: Peak Enhancement Intensity; TPEI: Time to PEI; MITR: Maximum Intensity Time ratio (PEI/TPEI) MGUS: monoclonal gammapathy of undetermined significance; SMM: smoldering multiple myeloma; Sympto: symptomatic multiple myeloma

**Fig. 4:**
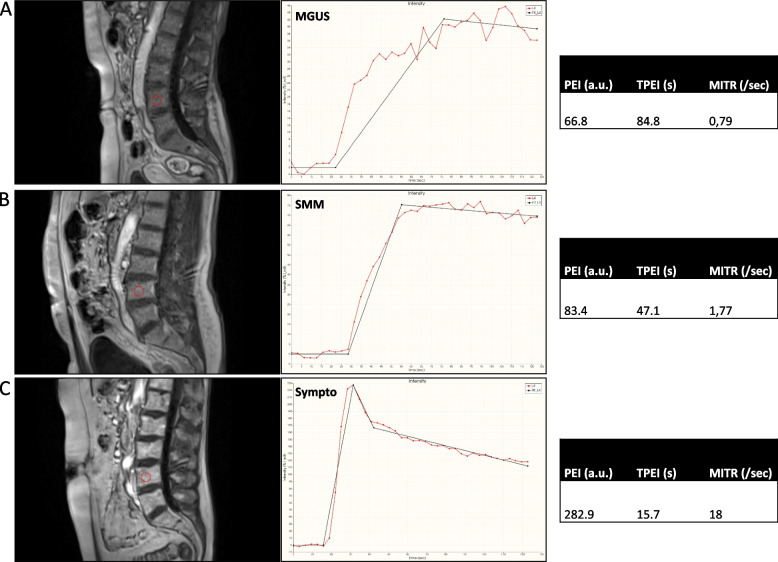
Three different patients and their corresponding time-signal intensity curves-derived Peak Enhancement Intensity (PEI)/ Time to PEI (TPEI)/ MITR (Maximum Intensity Time ratio) values (**A** MGUS: monoclonal gammapathy of undetermined significance. **B** SMM: smoldering multiple myeloma. **C** Sympto: symptomatic multiple myeloma)

Of all the DCE-MRI-based parameters extracted from the TIC, three were significantly different between the different stages of monoclonal plasma cell disease (Fig. 3). Arbitrary PEI values (mean ± standard deviation) were significantly higher in the symptomatic MM group (99 ± 48) than in the SMM (72 ± 37, *p* = 0.03) and MGUS (62 ± 24, *p* < 10^–4^) groups. TPEI mean duration was significantly higher in MGUS group (53 s ± 28) than in SMM (34 s ± 18, *p* = 0.02) and symptomatic MM (32 s ± 24, *p* < 10^–3^) groups. The maximum intensity time ratio (MITR) was the division of these two variables (PEI/TPEI) and the only parameter significantly different between all monoclonal plasma cell disease stages with a continuous increase from MGUS (1.62 s^−1^ ± 1.12) to SMM (2.76 s^−1^ ± 2.59) and finally symptomatic MM (4.93 s^−1^ ± 4.37). Figure 4 illustrates the difference of PEI, TPEI and MITR values along the spectrum of plasma cell disorders with three representative examples.

### Correlation between DCE-MRI-based parameters and diffusion-weighted MRI/PET-based parameters/biological markers

**Fig. 5:**
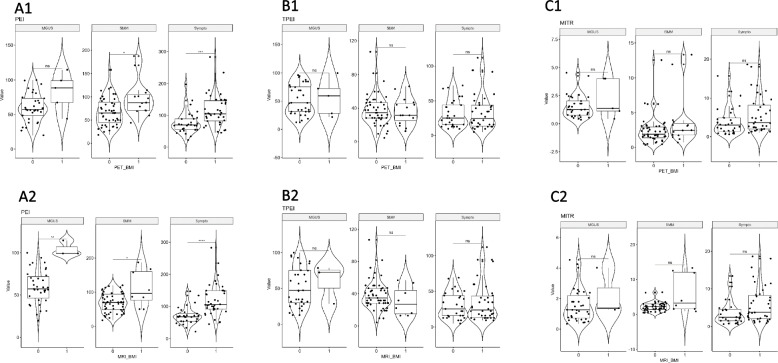
DCE-MRI-based PEI (**A**)/TPEI (**B**)/MITR (**C**) semi-quantitative parameters in different monoclonal plasma cell disease stages according to diffuse bone marrow involvement (BMI) status either by PET or by MRI (0 means absence of diffuse BMI, 1 means presence of diffuse BMI). NS: No significant; “*”: p < 0,05; “**”: p < *10*^*–2*^; “***”: p < *10*^*–3*^; “****”: p < *10*^*–4*^*)*. MGUS: monoclonal gammapathy of undetermined significance; SMM: smoldering multiple myeloma; Sympto: symptomatic multiple myeloma

**Fig. 6:**
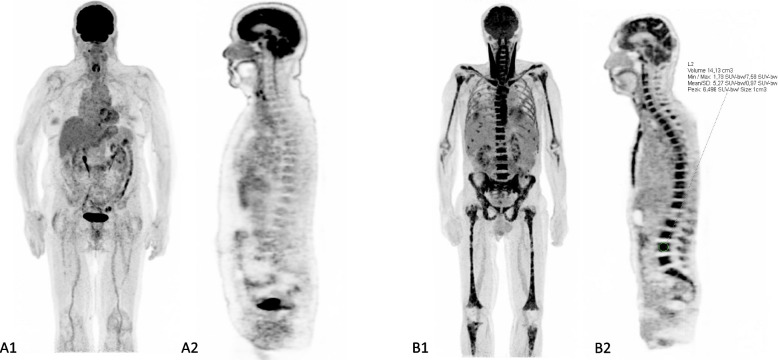
Patient with monoclonal gammapathy of undetermined significance (**A**) without diffuse bone marrow involvement (no significant uptake) on maximum intensity projection (MIP, A1) and sagittal (A2) positron emission tomography (PET) images. Patient with symptomatic multiple myeloma (**B**) and diffuse bone marrow involvement (diffuse uptake higher than liver background uptake) on MIP (B1) and sagittal (B2) PET images with maximum standardized uptake value (SUV_max_): 7.58 inside L4 vertebral body

Among the DCE-MRI-based parameters that differed significantly between all stages of monoclonal plasma cell disease, PEI values were significantly higher in patients with diffuse BMI on either PET or MRI images (Fig. 5 A1/A2) in all stages of monoclonal plasma cell disease. TPEI (Fig. 5 B1/B2) and MITR (Fig. 5 C1/C2) values were approximately the same (no significant difference) between patients with diffuse BMI on either PET (Fig. 6) or MRI images and patients without diffuse BMI (Fig. 6).

**Fig. 7:**
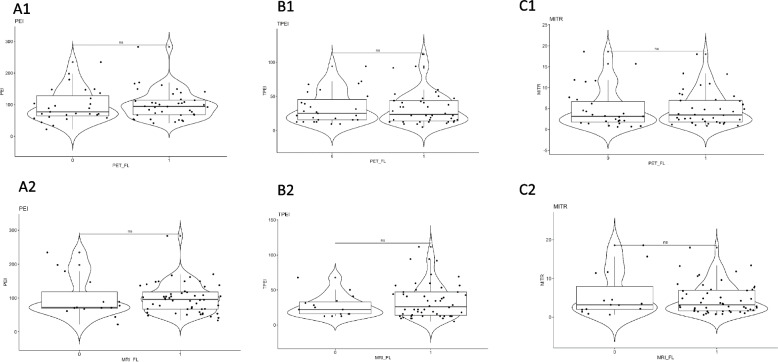
DCE-MRI-based PEI (**A**)/TPEI (**B**)/MITR (**C**) semi-quantitative parameters in symptomatic multiple myeloma disease stage according to focal lesion (FL) status either by PET or by MRI (0 means absence of FL, 1 means presence of FL. NS: no significant; “*”: p < 0,05; “**”: p < *10*^*–2*^; “***”: p < *10*^*–3*^; “****”: p < *10*^*–4*^*)*

PEI values were not significantly higher in patients with FL(s) on PET or MRI images (Fig. 7 A1/A2) than in patients without in the SMM and symptomatic MM groups. Similarly, TPEI (Fig. 7 B1/B2) and MITR (Fig. 7 C1/C2) values were not significantly different between patients with FL(s) on PET or MRI images and those without in the SMM and symptomatic MM groups.

No significant correlation was found between PEI, TPEI or MITR values and conventional biological markers (medullary monoclonal plasma cell percentage, serum concentration of monoclonal Ig, serum involved/uninvolved free-light chains ratio values, hemoglobin values, renal clearance values). The best (moderate) correlation was found between PEI and monoclonal medullary plasma cell percentage values (r = 0.31, *p* < 10^–6^).

## Discussion

We report here for the first time in a large prospective cohort data concerning bone marrow DCE-MRI in all monoclonal plasma cell disease stages and its relationship with conventional MRI and PET-based biomarkers in a hybrid simultaneous whole body-2-[18F]FDG-PET/MRI imaging approach.

DCE-MRI by a kinetic analysis of the accumulation and distribution of contrast medium (Gadolinium-DTPA) in vivo in the bone marrow in T1-weighted sequences covering the lumbar spine allows the study of microcirculation/angiogenesis [7, 16, 17]. In this study, in three significant groups of MGUS/SMM/symptomatic MM patients according to IMWG criteria, we showed that different DCE-MRI-based parameters (PEI, TPEI and their ratio MITR) could significantly differentiate all monoclonal plasma cell disease stages. PEI values increased and TPEI values decreased progressively along the spectrum of plasma cell disorders, from MGUS stage to symptomatic myeloma. Statistical significance was almost reached for PEI values between MGUS and SMM groups (*p* = 0.1) and for TPEI values between SMM and symptomatic MM groups (*p* = 0.056) and in these comparisons the second parameter was significantly different highlighting the complementarity of PEI and TPEI. Furthermore, the MITR of these two parameters (PEI/TPEI) allowed to increase the level of significance between each group and was the only parameter significantly different between all monoclonal plasma cell disease stages with a continuous increase from MGUS to SMM and finally symptomatic MM.

These results are in line with what has previously been demonstrated in an invasive way [17]. In this study, bone marrow angiogenesis was invetigated in bone marrow samples obtained from biopsies by immunohistochemical staining for CD34 to identify microvessels. Micro-vessel density was then determined and increased continuously from MGUS to SMM, symptomatic MM and finally relapsed disease. Our results suggest that DCE-MRI and especially PEI, TPEI, MITR biomarkers could be considered as non-invasive surrogates for the assessment of bone marrow angiogenesis. PEI and TPEI could reflect micro-vessel density and permeability and tumor tissue is more vascularized than normal tissue with the formation of new blood vessels with thin and permeable walls resulting in accumulation of gadolinium-DTPA in the interstitial space [18].

In this study, we explored for the first time the relationship between DCE-MRI-based parameters and diffusion-weighted MRI/PET biomarkers in a hybrid simultaneous whole body-2-[18F] FDG-PET/MRI imaging approach. Among the DCE-MRI-based parameters that differed significantly between all stages of monoclonal plasma cell disease, PEI values were significantly higher in patients with diffuse BMI (either on PET or MRI images) than in those without reflecting a correlation between these parameters. Thus, PEI seems to be correlated with bone marrow cellularity as measured by ADC (hypercellularity in diffuse BMI on MRI images) and clonal plasma cell morphology as measured by SUV_max_ (high bone marrow’s SUV_max_ value indicates low differentiation of clonal plasma cells [13]). However, TPEI values were approximately the same between patients with diffuse BMI on either PET or MRI images and those without diffuse BMI, meaning that these parameters are unlikely to be correlated. The speed of bone marrow perfusion thus reflects another biological phenomenon that bone marrow cellularity or clonal plasma cell morphology. PEI and TPEI values were not significantly different between patients with FL(s) on PET or MRI images and those without in symptomatic MM group meaning that bone marrow angiogenesis was not correlated with MM-related bone disease in this study. The best (moderate) correlation found between PEI or TPEI values and biological conventional markers was between PEI and medullary monoclonal plasma cells percentage values (r = 0.31, *p* < 10^–6^), which is consistent with the correlation between PEI and bone marrow cellularity represented by ADC described above.

We acknowledge that DCE-MRI post-processing image analysis using a two-compartment pharmacokinetic model, which allows absolute quantification of microcirculation/angiogenesis, may be more physiological and accurate. However, we chose a semi-quantitative method for DCE-MRI analysis as previously described [11, 12]. In this study, DCE-MRI was performed at the end of a whole-body 2-[18F]FDG-PET/MRI scan of approximately one-hour duration for the assessment of multiple myeloma, so we chose to perform the fastest DCE-MRI protocol. The TICs were normalized to automatically derive semi-quantitative parameters. This method is relatively easy to use and applicable in routine clinical practice. The TIC reflects the passage of contrast agent from the intravascular space to the interstitial space, and TIC-based semi-quantitative parameters such as PEI or TPEI provide valuable information on the degree and speed of relative signal enhancement and could therefore represent relative blood volume and vessel wall permeability. We acknowledge that TIC-derived parameters are semi-quantitative and therefore sensitive to variation between acquisition protocols and dependent on the amount of contrast agent and scan duration, but the reproducibility of these TIC-derived parameters could be increased with a standardized DCE-MRI protocol as proposed in this and previous work.Several studies have shown that DCE-MRI-based parameters can also provide important complementary data at baseline in symptomatic MM for predicting new lumbar vertebral fractures and clinical outcome [9] and in SMM patients for predicting progression to active MM disease [10]. Follow-up of patients enrolled in this study will allow a prospective comparison of the prognostic value and complementarity of baseline DCE-MRI parameters with more conventional MRI and PET-based biomarkers extracted from hybrid simultaneous WB-2-[18F]FDG-PET/MRI imaging in all plasma cell disease stages.

## Conclusions

Bone marrow DCE-MRI-based PEI and TPEI parameters allow to distinguish all monoclonal plasma cell disease stages and are unequally correlated with other diffusion-weighted MRI and PET-based biomarkers in a hybrid simultaneous WB-2-[18F]FDG-PET/MRI imaging approach.

## Data Availability

The datasets used and/or analysed during the current study are available from the corresponding author on reasonable request.

## Abbreviations

ADC: Apparent diffusion coefficient
BMI: Bone marrow involvement
DCE-MRI: Dynamic contrast enhanced-MRI
FL: Focal bone lesion
IMWG: International myeloma working group
MDE: Myeloma defining event
MGUS: Monoclonal gammapathy of undetermined significance
MITR: Maximum Intensity Time ratio (PEI/TPEI)
MM: Multiple myeloma
PEI: Peak enhancement intensity
ROI: Region of interest
SMM: Smoldering multiple myeloma
SUV_max_: Maximal standardized uptake value
TIC: Time-signal intensity curve
TPEI: Time to PEI
WB-2-[18F]FDG-PET/MRI: Whole body-2-18F-fluorodeoxyglucose-positron emission tomography coupled with magnetic resonance imaging
